# Reverses of Ando’s and Hölder–McCarty’s inequalities

**DOI:** 10.1186/s13660-018-1758-z

**Published:** 2018-07-05

**Authors:** Monire Hajmohamadi, Rahmatollah Lashkaripour, Mojtaba Bakherad

**Affiliations:** Department of Mathematics, Faculty of Mathematics, University of Sistan and Baluchestan, Zahedan, Iran

**Keywords:** 47A63, 15A64, Positive operator, Young inequality, Hölder–McCarthy’s inequality, Ando’s inequality

## Abstract

In this paper, we give some reverse-types of Ando’s and Hölder–McCarthy’s inequalities for positive linear maps, and positive invertible operators. For this purpose, we use a recently improved Young inequality and its reverse.

## Introduction and preliminaries

Let $\mathcal{B}(\mathcal{H})$ be the $C^{*}$-algebra of all bounded linear operators on a complex Hilbert space $\mathcal{H}$ with the operator norm $\Vert \cdot \Vert $ and the identity *I*; also $\mathcal{M} _{n}(\mathcal{C})$ denotes the space of all $n\times n$ complex matrices. For an operator $A \in \mathcal{B}(\mathcal{H})$, we write $A\geq 0$ if *A* is positive, and $A>0$ if *A* is positive invertible. For $A, B \in \mathcal{B}(\mathcal{H})$, we say $A\geq B$ if $A-B\geq 0$. The Gelfand map $f(t)\mapsto f(A)$ is an isometrical ∗-isomorphism between the $C^{*}$-algebra $C(\operatorname {sp}(A))$ of continuous functions on the spectrum $\operatorname {sp}(A)$ of a selfadjoint operator *A* and the $C^{*}$-algebra generated by *A* and *I*. A linear map Φ on $\mathcal{B}(\mathcal{H})$ is positive if $\Phi (A) \geq 0$ whenever $A\geq 0$. It is said to be unital if $\Phi (I)=I$. A continuous function $f:J\rightarrow \mathcal{R}$ is operator concave if

$$\begin{aligned} f\bigl(\alpha A+(1-\alpha )B\bigr) \geq \alpha f(A)+(1-\alpha )f(B) \end{aligned}$$

for all selfadjoint operators $A, B\in \mathcal{B}(\mathcal{H})$ with spectra in *J* and all $\alpha \in [0,1]$.

The well-known Young inequality says that, for positive real numbers *a*, *b* and $0\leq t \leq 1$, we have $a^{t}b^{1-t}\leq t a+(1-t)b$. Refinements and reverses of this inequality are proven in [2, 9, 14–16] and the references therein. Also Kittaneh et al. in [10] obtained the following improvement of the Young inequality for any positive definite matrices $A,B\in \mathcal{M}_{n}(\mathcal{C})$:

1 $$\begin{aligned} A^{1-t}B^{t}+r(A+B-2A\sharp B)\leq (1-t)A+t B \leq A^{1-t}B^{t}+R(A+B-2A \sharp B), \end{aligned}$$

where $t\in [0,1]$, $r=\min \{t,1-t\}$ and $R=\max \{t,1-t\}$.

Zhao et al. [19] obtained a refinement of Young’s inequality and its reverse as follows:

(i) for $0< t\leq \frac{1}{2}$,
  2 $$\begin{aligned} &r_{0}\bigl(\sqrt[4]{ab}-\sqrt{a}\bigr)^{2}+r( \sqrt{a}-\sqrt{b})^{2} +a ^{1-t}b^{t} \\ &\quad \leq (1-t)a+tb \\ &\quad \leq R(\sqrt{a}-\sqrt{b})^{2}-r_{0}\bigl(\sqrt[4]{ab}- \sqrt{b}\bigr)^{2}+a ^{1-t}b^{t}; \end{aligned}$$
(ii) for $\frac{1}{2}< t<1$,
  $$\begin{aligned} &r_{0}\bigl(\sqrt[4]{ab}-\sqrt{b}\bigr)^{2}+R(\sqrt{a}- \sqrt{b})^{2} +a ^{1-t}b^{t}\\ &\quad \leq (1-t)a+tb \\ &\quad \leq r(\sqrt{a}-\sqrt{b})^{2}-r_{0}\bigl(\sqrt[4]{ab}- \sqrt{a}\bigr)^{2}+a ^{1-t}b^{t}, \end{aligned}$$

where $r=\min \{t,1-t\}$, $R=\max \{t,1-t\}$ and $r_{0}=\min \{2r,1-2r \}$.

Sababheh et al. [15, 16] established some refinements and reverses of Young’s inequality as follows:

(i) for $0\leq t\leq \frac{1}{2}$,
  3 $$\begin{aligned} S_{N}(t;a,b)\leq t a+(1-t)b-a^{t} b^{1-t}\leq (1-t) (\sqrt{a}- \sqrt{b})^{2}-S_{N}(2t; \sqrt{ab},a); \end{aligned}$$
(ii) for $\frac{1}{2}\leq t\leq 1$,
  $$\begin{aligned} S_{N}(t;a,b)\leq t a+(1-t)b-a^{t} b^{1-t}\leq t( \sqrt{a}-\sqrt{b})^{2}-S _{N}(2-2t;\sqrt{ab},b), \end{aligned}$$

where

$$\begin{aligned} S_{N}(t;a,b)=\sum_{j=1}^{N}s_{j}(t) \bigl(\sqrt[2^{j}]{b^{2^{j-1}-k_{j}}a ^{k_{j}}}- \sqrt[2^{j}]{a^{k_{j}+1}b^{2^{j-1}-k_{j}-1}} \bigr)^{2}, \end{aligned}$$

$s_{j}(t)= ((-1)^{r_{j}}2^{j-1}t+(-1)^{r_{j}+1}[ \frac{r_{j}+1}{2}] )$, $r_{j}=[2^{j}t]$ and $k_{j}=[2^{j-1}t]$. Here $[x]$ is the greatest integer less than or equal to *x*.

Let $A, B\in \mathcal{B}(\mathcal{H})$ be positive. The operator *t*-weighted arithmetic, geometric, and harmonic means of operators *A*, *B* are defined by $A\nabla_{t}B=(1-t)A+tB$, $A\sharp_{t}B=A^{ \frac{1}{2}}(A^{-\frac{1}{2}}B A^{-\frac{1}{2}})^{t}A^{\frac{1}{2}}$ and $A!_{t}B=((1-t)A^{-1}+tB^{-1})^{-1}$, respectively. In particular, for $t =\frac{1}{2}$ we get the usual operator arithmetic mean ∇, the geometric mean ♯ and the harmonic mean !.

## Results and discussion

For positive real numbers $a_{i}$ and $b_{i} $ ($i=1,2,\ldots,n$) the Hölder inequality states that

4 $$\begin{aligned} \sum_{i=1}^{n}a_{i}^{1/p}b_{i}^{1/q} \leq \Biggl( \sum_{i=1}^{n}a_{i} \Biggr) ^{1/p} \Biggl( \sum_{i=1}^{n}b_{i} \Biggr) ^{1/q} \end{aligned}$$

for $p,q>1$ such that $\frac{1}{p}+\frac{1}{q}=1$. If $p=q=2$ in (4), then we get the Cauchy–Schwarz inequality. The Hölder inequality for positive operators $A_{i}$ and $B_{i} $ ($i=1,2,\ldots,n$) is

$$\begin{aligned} \sum_{i=1}^{n}A_{i} \sharp_{t} B_{i}\leq \Biggl( \sum _{i=1}^{n}A_{i} \Biggr) \sharp_{t} \Biggl( \sum_{i=1}^{n}B_{i} \Biggr) , \end{aligned}$$

where $0\leq t\leq 1$. In the case $t=\frac{1}{2}$, we get the operator Cauchy–Schwarz inequality. For further information as regards the Hölder and Cauchy–Schwarz inequalities we refer the reader to [3–5, 11, 12, 20] and the references therein. Ando [1] proved that if Φ is a positive linear map, then for positive operators $A, B\in \mathcal{B}(\mathcal{H})$ and $t\in [0,1]$, we have

$$\begin{aligned} \Phi (A\sharp_{t} B)\leq \Phi (A)\sharp_{t}\Phi (B). \end{aligned}$$

Recently, some authors presented several reverse-types of Ando’s inequality (see [13, 17]).

The Hölder–McCarthy’s inequality says that for any positive operator *A* and any unit vector $x\in \mathcal{H}$, we have

5 $$\begin{aligned} \bigl\langle A^{t}x,x\bigr\rangle \leq \langle Ax,x \rangle^{t}, \quad 0\leq t\leq 1. \end{aligned}$$

Furuta [8] showed that this inequality is equivalent to Young’s inequality.

## Conclusions

In this paper, we establish a reverse of Ando’s inequality for positive (non-unital) linear maps and positive definite matrices by using an inequality due to Sababheh. We obtain some reverses of the matrix Hölder and Cauchy–Schwarz inequalities and a reverse of inequality (5) for $t\in (0,\frac{1}{2}]$ as follows:

$$\begin{aligned} &\langle Tx,x \rangle^{t}-\bigl\langle T^{t}x,x\bigr\rangle \\ & \quad \leq 2R \bigl( \langle Tx,x\rangle^{\frac{1}{2}}-\bigl\langle T^{ \frac{1}{2}}x,x\bigr\rangle \bigr) -r_{0} \bigl( \bigl\langle T^{\frac{1}{2}}x,x \bigr\rangle + \langle Tx,x\rangle^{\frac{1}{2}}-2\bigl\langle T^{\frac{1}{4}}x,x \bigr\rangle \langle Tx,x\rangle^{\frac{1}{4}} \bigr) . \end{aligned}$$

## Methods

We use the properties of inner product and the inequalities obtained in [16] and [19].

## Main results

To prove our first result, we need the following lemmas.

### Lemma 1

([16])

*Let* $A, B\in \mathcal{M}_{n}(\mathcal{C})$ *be positive definite matrices and* $t\in [0,1]$. *Then*

6 $$\begin{aligned} \sum_{j=1}^{N}s_{j}(t) ( A\sharp_{\alpha_{j}(t)}B+A \sharp_{2^{1-j}+\alpha_{j}(t)}B-2A\sharp_{2^{-j}+\alpha_{j}(t)}B ) +A \sharp_{t}B\leq A\nabla_{t}B, \end{aligned}$$

*where* $\alpha_{j}(t)=\frac{k_{j}(t)}{2^{j-1}}$.

For $N=2$, we have the following lemma, which is shown in [19] for positive invertible operators.

### Lemma 2

*Let* $A, B\in \mathcal{B}(\mathcal{H})$ *be positive invertible operators and* $t\in [0,1]$.

(i) *If* $0< t\leq \frac{1}{2}$, *then*
  7 $$\begin{aligned} r_{0}(A\sharp B-2A\sharp_{\frac{1}{4}}B+A)+2t(A\nabla B-A\sharp B)+A \sharp_{t}B\leq A\nabla_{t}B. \end{aligned}$$
(ii) *If* $\frac{1}{2}< t<1$, *then*
  8 $$\begin{aligned} r_{0}(A\sharp B-2A\sharp_{\frac{3}{4}}B+B)+2(1-t) (A \nabla B-A\sharp B)+A \sharp_{t}B\leq A\nabla_{t}B, \end{aligned}$$
  *where* $r=\min \{\nu ,1-\nu \}$ *and* $r_{0}=\min \{2r,1-2r\}$.

### Lemma 3

([16])

*Let* $A, B\in \mathcal{M}_{n}(\mathcal{C})$ *be positive definite matrices and* $t\in [0,1]$.

(i) *If* $0\leq t\leq \frac{1}{2}$, *then*
  9 $$\begin{aligned} A\nabla_{t}B&\leq A\sharp_{t}B+2(1-t) (A \nabla B-A\sharp B) \\ &\quad {} -\sum_{j=1}^{N}s_{j}(2t) ( A \sharp_{1-\beta_{j}(t)}B+A \sharp_{1+2^{-j}-\beta_{j}(t)}B-2A\sharp_{1-2^{-j-1}-\beta_{j}(t)}B ) . \end{aligned}$$
(ii) *If* $\frac{1}{2}\leq t\leq 1$, *then*
  $$\begin{aligned} A\nabla_{t}B &\leq A\sharp_{t}B+2t(A\nabla B-A\sharp B) \\ & \quad {} -\sum_{j=1}^{N}s_{j}(2-2t) ( A \sharp_{\gamma_{j}(t)}B+A \sharp_{\gamma_{j}(t)+2^{1-j}}B-2A\sharp_{\gamma_{j}(t)+2^{-j}}B ) , \end{aligned}$$
  *where* $\beta_{j}(t)=2^{-j}k_{j}(2t)$ *and* $\gamma_{j}(t)=2^{1-j}k _{j}(2-2t)$.

### Remark 4

By using functional calculus and numerical inequalities in [10, 16], we can extend inequality (1), Lemmas 1 and 3 for positive invertible operators.

For $N=2$, we have the following lemma, which is shown in [19] for positive invertible operators.

### Lemma 5

*Let* $A, B\in \mathcal{B}(\mathcal{H})$ *be positive invertible operators and* $t\in [0,1]$.

(i) *If* $0< t\leq \frac{1}{2}$, *then*
  10 $$\begin{aligned} A\nabla_{t}B \leq A\sharp_{t}B+2(1-t) (A\nabla B-A\sharp B)-r_{0}(A \sharp B-2A\sharp_{\frac{3}{4}}B+B). \end{aligned}$$
(ii) *If* $\frac{1}{2}< t<1$, *then*
  11 $$\begin{aligned} A\nabla_{t}B \leq A\sharp_{t}B+2t(A\nabla B-A \sharp B)-r_{0}(A\sharp B-2A\sharp_{\frac{1}{4}}B+A), \end{aligned}$$
  *where* $r=\min \{\nu ,1-\nu \}$ *and* $r_{0}=\min \{2r,1-2r\}$.

Now, we obtain a reverse of Ando’s inequality for positive invertible operators as follows.

### Theorem 6

*Let* $A, B\in \mathcal{B}(\mathcal{H})$ *be positive invertible operators*, Φ *be a positive linear map and* $t\in [0,1]$.

(i) *If* $0\leq t\leq \frac{1}{2}$, *then*
  12 $$\begin{aligned} & \Phi (A) \sharp_{t}\Phi (B)-\Phi (A \sharp_{t}B) \\ &\quad \leq 2R\biggl(\Phi (A)\sharp \Phi (B)-\Phi (A\sharp B)+ \frac{1}{2}\bigl(\Phi (A)+\Phi (B)-2\Phi (A) \sharp \Phi (B)\bigr)\biggr) \\ &\quad \quad {} -\sum_{j=1}^{N}s_{j}(2t) \bigl( \Phi (A\sharp_{1-\beta_{j}(t)}B)+ \Phi (A\sharp_{1+2^{-j}-\beta_{j}(t)}B)- 2\Phi (A \sharp_{1-2^{-j-1}-\beta_{j}(t)}B) \bigr) \\ &\quad \quad {} -\sum_{j=1}^{N}s_{j}(t) \bigl( \Phi (A)\sharp_{\alpha_{j}(t)}\Phi (B)+ \Phi (A)\sharp_{2^{1-j}+\alpha_{j}(t)}\Phi (B) \\ &\quad \quad {}-2\Phi (A) \sharp_{2^{-j}+\alpha_{j}(t)}\Phi (B) \bigr) . \end{aligned}$$
(ii) *If* $\frac{1}{2}\leq t\leq 1$, *then*
  13 $$\begin{aligned} &\Phi (A) \sharp_{t}\Phi (B)-\Phi (A \sharp_{t}B) \\ &\quad \leq 2R\biggl(\Phi (A)\sharp \Phi (B)-\Phi (A\sharp B)+ \frac{1}{2}\bigl(\Phi (A)+\Phi (B)-2\Phi (A) \sharp \Phi (B)\bigr)\biggr) \\ &\quad \quad {} -\sum_{j=1}^{N}s_{j}(2-2t) \bigl( \Phi (A\sharp_{\gamma_{j}(t)}B)+ \Phi (A\sharp_{\gamma_{j}(t)+2^{1-j}}B)-2\Phi (A \sharp_{\gamma_{j}(t)+2^{-j}}B) \bigr) \\ &\quad \quad {} -\sum_{j=1}^{N}s_{j}(t) \bigl( \Phi (A)\sharp_{\alpha_{j}(t)}\Phi (B)+ \Phi (A)\sharp_{2^{1-j}+\alpha_{j}(t)}\Phi (B) \\ &\quad \quad {}-2\Phi (A) \sharp_{2^{-j}+\alpha_{j}(t)}\Phi (B) \bigr), \end{aligned}$$
  *where* $\alpha_{j}(t)=\frac{k_{j}(t)}{2^{j-1}}$, $\beta_{j}(t)=2^{-j}k _{j}(2t)$, $\gamma_{j}(t)=2^{1-j}k_{j}(2-2t)$ *and* $R=\max \{t,1-t\}$.

### Proof

The proof of inequality (13) is similar to the proof of inequality (12). Thus, we only prove inequality (12).

Let $0\leq t\leq \frac{1}{2}$. Applying inequalities (10) and (9), we have

14 $$\begin{aligned} &\sum_{j=1}^{N}s_{j}(t) (A\sharp_{\alpha_{j}(t)}B+A \sharp_{2^{1-j}+\alpha_{j}(t)}B-2A\sharp_{2^{-j}+\alpha_{j}(t)}B) \\ &\quad \leq A\nabla_{t} B-A\sharp_{t}B \\ &\quad \leq 2R(A\nabla B-A\sharp B) -\sum_{j=1}^{N}s_{j}(2t) ( A \sharp_{1-\beta_{j}(t)}B+A\sharp_{1+2^{-j}-\beta_{j}(t)}B-2A \sharp_{1-2^{-j-1}-\beta_{j}(t)}B ) . \end{aligned}$$

Now, using the positive linear map Φ on (14), we get

15 $$\begin{aligned} &\sum_{j=1}^{N} s_{j}(t) \bigl( \Phi (A\sharp_{\alpha_{j}(t)}B)+\Phi (A \sharp_{2^{1-j}+\alpha_{j}(t)}B)-2\Phi (A\sharp_{2^{-j}+\alpha_{j}(t)}B) \bigr) +\Phi (A \sharp_{t}B) \\ &\quad \leq \Phi (A)\nabla_{t} \Phi (B) \\ &\quad \leq 2R \bigl( \Phi (A)\nabla \Phi (B)-\Phi (A\sharp B) \bigr) +\Phi (A \sharp_{t}B) \\ & \quad\quad {} -\sum_{j=1}^{N}s_{j}(2t) \bigl( \Phi (A\sharp_{1-\beta_{j}(t)}B)+ \Phi (A\sharp_{1+2^{-j}-\beta_{j}(t)}B)-2\Phi (A \sharp_{1-2^{-j-1}-\beta_{j}(t)}B) \bigr) . \end{aligned}$$

Moreover, if we replace *A* and *B* by $\Phi (A)$ and $\Phi (B)$ in inequality (14), respectively, then

16 $$\begin{aligned} &\sum_{j=1}^{N}s_{j}(t) \bigl( \Phi (A)\sharp_{\alpha_{j}(t)}\Phi (B)+ \Phi (A)\sharp_{2^{1-j}+ \alpha_{j}(t)}\Phi (B)-2\Phi (A) \sharp_{2^{-j}+\alpha_{j}(t)}\Phi (B) \bigr) \\ &\quad\quad {} +\Phi (A)\sharp_{t}\Phi (B) \\ &\quad \leq \Phi (A)\nabla_{t}\Phi (B) \\ &\quad \leq 2R \bigl( \Phi (A)\nabla \Phi (B)-\Phi (A\sharp B) \bigr) +\Phi (A) \sharp_{t}\Phi (B) \\ &\quad\quad {} -\sum_{j=1}^{N}s_{j}(2t) \bigl( \Phi (A)\sharp_{1-\beta_{j}(t)}\Phi (B)+ \Phi (A)\sharp_{1+2^{-j}-\beta_{j}(t)}\Phi (B) \\ &\quad\quad {}-2\Phi (A) \sharp_{1-2^{-j-1}-\beta_{j}(t)}\Phi (B) \bigr) . \end{aligned}$$

From the first inequality of (16) and the second inequality of (15), we have

$$\begin{aligned} &\sum_{j=1}^{N} s_{j}(t) \bigl( \Phi (A)\sharp_{\alpha_{j}(t)}\Phi (B)+ \Phi (A)\sharp_{2^{1-j}+ \alpha_{j}(t)}\Phi (B)-2\Phi (A) \sharp_{2^{-j}+\alpha_{j}(t)}\Phi (B) \bigr) \\ & \quad\quad {} +\Phi (A)\sharp_{t}\Phi (B) \\ & \quad \leq \Phi (A)\nabla_{t}\Phi (B) \\ &\quad \leq 2R\bigl(\Phi (A)\nabla \Phi (B)-\Phi (A\sharp B)\bigr)+\Phi (A \sharp_{t}B) \\ & \quad\quad {} -\sum_{j=1}^{N}s_{j}(2t) \bigl( \Phi (A\sharp_{1-\beta_{j}(t)}B)+ \Phi (A\sharp_{1+2^{-j}-\beta_{j}(t)}B)-2\Phi (A \sharp_{1-2^{-j-1}-\beta_{j}(t)}B) \bigr) , \end{aligned}$$

which implies that

$$\begin{aligned} &\sum_{j=1}^{N} s_{j}(t) \bigl( \Phi (A)\sharp_{\alpha_{j}(t)}\Phi (B)+ \Phi (A)\sharp_{2^{1-j}+ \alpha_{j}(t)}\Phi (B)-2\Phi (A) \sharp_{2^{-j}+\alpha_{j}(t)}\Phi (B) \bigr) \\ & \quad\quad {} +\Phi (A)\sharp_{t}\Phi (B) \\ & \quad \leq 2R \bigl( \Phi (A)\nabla \Phi (B)-\Phi (A\sharp B) \bigr) +\Phi (A \sharp_{t}B) \\ &\quad\quad {} -\sum_{j=1}^{N}s_{j}(2t) \bigl( \Phi (A\sharp_{1-\beta_{j}(t)}B)+ \Phi (A\sharp_{1+2^{-j}-\beta_{j}(t)}B)-2\Phi (A \sharp_{1-2^{-j-1}-\beta_{j}(t)}B) \bigr) . \end{aligned}$$

Therefore, applying inequality (1), we get

$$\begin{aligned} &\Phi (A) \sharp_{t}\Phi (B)-\Phi (A\sharp_{t}B) \\ &\quad \leq 2R \bigl(\Phi (A)\nabla \Phi (B)-\Phi (A\sharp B)\bigr) \\ & \quad \quad {} -\sum_{j=1}^{N}s_{j}(2t) \bigl( \Phi (A\sharp_{1-\beta_{j}(t)}B)+ \Phi (A\sharp_{1+2^{-j}-\beta_{j}(t)}B)- 2\Phi (A \sharp_{1-2^{-j-1}-\beta_{j}(t)}B) \bigr) \\ &\quad \quad {} -\sum_{j=1}^{N}s_{j}(t) \bigl( \Phi (A)\sharp_{\alpha_{j}(t)}\Phi (B)+ \Phi (A)\sharp_{2^{1-j}+\alpha_{j}(t)}\Phi (B)-2\Phi (A) \sharp_{2^{-j}+\alpha_{j}(t)}\Phi (B) \bigr) \\ & \quad \leq 2R\biggl(\Phi (A)\sharp \Phi (B)-\Phi (A\sharp B)+\frac{1}{2}\bigl( \Phi (A)+ \Phi (B)-2\Phi (A)\sharp \Phi (B)\bigr)\biggr) \\ & \quad \quad {} -\sum_{j=1}^{N}s_{j}(2t) \bigl( \Phi (A\sharp_{1-\beta_{j}(t)}B)+ \Phi (A\sharp_{1+2^{-j}-\beta_{j}(t)}B)- 2\Phi (A \sharp_{1-2^{-j-1}-\beta_{j}(t)}B) \bigr) \\ &\quad \quad {} -\sum_{j=1}^{N}s_{j}(t) \bigl( \Phi (A)\sharp_{\alpha_{j}(t)}\Phi (B)+ \Phi (A)\sharp_{2^{1-j}+\alpha_{j}(t)}\Phi (B)-2\Phi (A) \sharp_{2^{-j}+\alpha_{j}(t)}\Phi (B) \bigr) . \end{aligned}$$

 □

Similarly for $N=2$ by applying Lemma 2 and Lemma 5, we can obtain a reverse of Ando’s inequality for positive invertible operators.

### Corollary 7

*Let* $A, B\in \mathcal{B}(\mathcal{H})$ *be positive invertible operators*, Φ *be a positive linear map and* $t\in [0,1]$.

(i) *If* $0< t\leq \frac{1}{2}$, *then*
  17 $$\begin{aligned} & \Phi (A)\sharp_{t}\Phi (B)-\Phi (A\sharp_{t}B) \\ &\quad \leq 2R\biggl(\Phi (A)\sharp \Phi (B)-\Phi (A\sharp B)+\frac{1}{2}\bigl( \Phi (A)+\Phi (B)-2\Phi (A) \sharp \Phi (B)\bigr)\biggr) \\ &\quad \quad {} -r_{0}\bigl(\Phi (A\sharp B)+\Phi (B)-2\Phi (A\sharp_{\frac{3}{4}}B) \bigr) \\ &\quad \quad {} -r_{0}\bigl(\Phi (A)\sharp \Phi (B)+\Phi (A)-2\bigl(\Phi (A) \sharp_{ \frac{1}{4}}\Phi (B)\bigr)\bigr) \\ & \quad \leq 2R\biggl(\Phi (A)\sharp \Phi (B)-\Phi (A\sharp B)+\frac{1}{2}\bigl( \Phi (A)+ \Phi (B)-2\Phi (A)\sharp \Phi (B)\bigr)\biggr); \end{aligned}$$
(ii) *if* $\frac{1}{2}< t< 1$, *then*
  18 $$\begin{aligned} &\Phi (A)\sharp_{t}\Phi (B)-\Phi (A\sharp_{t}B) \\ &\quad \leq 2R\biggl(\Phi (A)\sharp \Phi (B)-\Phi (A\sharp B)+\frac{1}{2}\bigl( \Phi (A)+\Phi (B)-2\Phi (A) \sharp \Phi (B)\bigr)\biggr) \\ &\quad \quad {} -r_{0}\bigl(\Phi (A)\sharp \Phi (B)+\Phi (B)-2\bigl(\Phi (A) \sharp_{ \frac{3}{4}}\Phi (B)\bigr)\bigr) \\ &\quad \quad {} -r_{0}\bigl(\Phi (A\sharp B)+\Phi (A)-2\Phi (A\sharp_{\frac{1}{4}} B)\bigr) \\ &\quad \leq 2R\biggl(\Phi (A)\sharp \Phi (B)-\Phi (A\sharp B)+\frac{1}{2}\bigl( \Phi (A)+ \Phi (B)-2\Phi (A)\sharp \Phi (B)\bigr)\biggr), \end{aligned}$$
  *where* $r=\min \{t,1-t\}$, $R=\max \{t,1-t\}$ *and* $r_{0}=\min \{2r,1-2r \}$.

We want to establish some inequalities for positive invertible operators.

### Theorem 8

*Let* $A, B\in \mathcal{B}(\mathcal{H})$ *be positive invertible*. *If* $t\in [0,1]$ *and* Φ, Ψ *are two unital positive linear maps*, *then for any unit vector* $x\in \mathcal{H}$

(i) *for* $0< t\leq \frac{1}{2}$,
  19 $$\begin{aligned} &2r \bigl( \bigl\langle \Phi (A)x,x\bigr\rangle \nabla \bigl\langle \Psi (B)x,x\bigr\rangle -\bigl\langle \Phi \bigl(A^{\frac{1}{2}}\bigr)x,x \bigr\rangle \bigl\langle \Psi \bigl(B^{1/2}\bigr)x,x \bigr\rangle \bigr) \\ &\quad\quad {} + r_{0} \bigl( \bigl\langle \Phi \bigl(A^{\frac{1}{2}}\bigr)x,x \bigr\rangle \bigr)\bigl\langle \Psi \bigl(B^{\frac{1}{2}}\bigr)x,x\bigr\rangle + \bigl\langle \Phi (A)x,x\bigr\rangle -2\bigl\langle \Phi \bigl(A^{\frac{3}{4}} \bigr)x,x\bigr\rangle \bigl\langle \Psi \bigl(B^{\frac{1}{4}}\bigr)x,x \bigr\rangle \bigr) \\ &\quad \leq (1-t)\bigl\langle \Phi (A)x,x\bigr\rangle +t\bigl\langle \Psi (B)x,x\bigr\rangle - \bigl\langle \Psi \bigl(B^{t}\bigr)x,x\bigr\rangle \bigl\langle \Phi \bigl(A^{1-t}\bigr)x,x\bigr\rangle \\ &\quad \leq 2R \bigl( \bigl\langle \Phi (A)x,x\bigr\rangle \nabla \bigl\langle \Psi (B)x,x \bigr\rangle -\bigl\langle \Phi \bigl(A^{\frac{1}{2}}\bigr)x,x\bigr\rangle \bigl\langle \Psi \bigl(B^{ \frac{1}{2}}\bigr)x,x\bigr\rangle \bigr) \\ &\quad\quad {} -r_{0} \bigl( \bigl\langle \Phi \bigl(A^{\frac{1}{2}}\bigr)x,x\bigr\rangle \bigl\langle \Psi \bigl(B ^{\frac{1}{2}}\bigr)x,x\bigr\rangle +\bigl\langle \Psi (B)x,x\bigr\rangle -2\bigl\langle \Phi \bigl(A^{\frac{1}{4}} \bigr)x,x\bigr\rangle \bigl\langle \Psi \bigl(B^{\frac{3}{4}}\bigr)x,x \bigr\rangle \bigr) ; \end{aligned}$$
(ii) *for* $\frac{1}{2}< t<1$,
  $$\begin{aligned} &R \bigl( \bigl\langle \Phi (A)x,x\bigr\rangle +\bigl\langle \Psi (B)x,x\bigr\rangle -2 \bigl\langle \Phi \bigl(A^{\frac{1}{2}}\bigr)x,x\bigr\rangle \bigl\langle \Psi \bigl(B^{1/2}\bigr)x,x \bigr\rangle \bigr) \\ & \quad\quad {} + r_{0} \bigl( \bigl\langle \Phi \bigl(A^{\frac{1}{2}}\bigr)x,x \bigr\rangle \bigr)\bigl\langle \Psi \bigl(B^{\frac{1}{2}}\bigr)x,x\bigr\rangle + \bigl\langle \Phi (A)x,x\bigr\rangle -2\bigl\langle \Phi \bigl(A^{\frac{1}{4}} \bigr)x,x\bigr\rangle \bigl\langle \Psi \bigl(B^{\frac{3}{4}}\bigr)x,x \bigr\rangle \bigr) \\ & \quad \leq (1-t)\bigl\langle \Phi (A)x,x\bigr\rangle +t\bigl\langle \Psi (B)x,x\bigr\rangle - \bigl\langle \Psi \bigl(B^{t}\bigr)x,x\bigr\rangle \bigl\langle \Phi \bigl(A^{1-t}\bigr)x,x\bigr\rangle \\ & \quad \leq r \bigl( \bigl\langle \Phi (A)x,x\bigr\rangle +\bigl\langle \Psi (B)x,x \bigr\rangle -2\bigl\langle \Phi \bigl(A^{\frac{1}{2}}\bigr)x,x\bigr\rangle \bigl\langle \Psi \bigl(B^{ \frac{1}{2}}\bigr)x,x\bigr\rangle \bigr) \\ &\quad\quad {} -r_{0} \bigl( \bigl\langle \Phi \bigl(A^{\frac{1}{2}}\bigr)x,x\bigr\rangle \bigl\langle \Psi \bigl(B ^{\frac{1}{2}}\bigr)x,x\bigr\rangle +\bigl\langle \Phi (A)x,x\bigr\rangle -2\bigl\langle \Phi \bigl(A^{\frac{3}{4}} \bigr)x,x\bigr\rangle \bigl\langle \Psi \bigl(B^{\frac{1}{4}}\bigr)x,x \bigr\rangle \bigr) , \end{aligned}$$

*where* $r=\min \{t,1-t\}$, $R=\max \{t,1-t\}$, $r_{0}=\min \{2r,1-2r\}$.

### Proof

The proof of part (ii) is similar to the proof of part (i). Thus we only prove (i).

Applying inequality (2) for any positive real numbers *k*, *s*, we have

20 $$\begin{aligned} &r(k+s-2\sqrt{ks})+ r_{0}\bigl(k^{\frac{1}{2}}s^{\frac{1}{2}}+k-2k^{ \frac{3}{4}}s^{\frac{1}{4}} \bigr) \\ &\quad \leq (1-t)k+ts-k^{1-t}s^{t} \\ &\quad \leq R(k+s-2\sqrt{ks})-r_{0}\bigl(k^{\frac{1}{2}}s^{\frac{1}{2}}+s-2k ^{\frac{1}{4}}s^{\frac{3}{4}}\bigr). \end{aligned}$$

Fix *s* in (20). Then applying functional calculus to the operator *A*, we have

21 $$\begin{aligned} & r\bigl(A+sI-2\sqrt{s}A^{\frac{1}{2}}\bigr)+ r_{0} \bigl(A^{\frac{1}{2}}s^{ \frac{1}{2}}+A-2A^{\frac{3}{4}}s^{\frac{1}{4}}\bigr) \\ & \quad \leq (1-t)A+tsI-s^{t}A^{1-t} \\ & \quad \leq R\bigl(A+sI-2\sqrt{s}A^{\frac{1}{2}}\bigr)-r_{0} \bigl(A^{\frac{1}{2}}s^{ \frac{1}{2}}+sI-2A^{\frac{1}{4}}s^{\frac{3}{4}}\bigr). \end{aligned}$$

If we apply the positive linear map Φ and inner product for $x\in {\mathcal{H}}$ with $\Vert x \Vert =1$ in inequality (21), we have

$$\begin{aligned} &r \bigl( \bigl\langle \Phi (A)x,x\bigr\rangle +s-2\sqrt{s}\bigl\langle \Phi \bigl(A^{ \frac{1}{2}}\bigr)x,x\bigr\rangle \bigr) \\ &\quad\quad {} + r_{0} \bigl( \bigl\langle \Phi \bigl(A^{\frac{1}{2}}\bigr)x,x \bigr\rangle \bigr)s^{ \frac{1}{2}}+\bigl\langle \Phi (A)x,x\bigr\rangle -2\bigl\langle \Phi \bigl(A^{ \frac{3}{4}}\bigr)x,x\bigr\rangle s^{\frac{1}{4}} \bigr) \\ &\quad \leq (1-t)\bigl\langle \Phi (A)x,x\bigr\rangle +ts-s^{t}\bigl\langle \Phi \bigl(A^{1-t}\bigr)x,x \bigr\rangle \\ &\quad \leq R \bigl( \bigl\langle \Phi (A)x,x\bigr\rangle +s-2\sqrt{s}\bigl\langle \Phi \bigl(A ^{\frac{1}{2}}\bigr)x,x\bigr\rangle \bigr) -r_{0} \bigl( \bigl\langle \Phi \bigl(A^{ \frac{1}{2}}\bigr)x,x\bigr\rangle s^{\frac{1}{2}}+s-2 \bigl\langle \Phi \bigl(A^{ \frac{1}{4}}\bigr)x,x\bigr\rangle s^{\frac{3}{4}} \bigr) . \end{aligned}$$

Now, using the functional calculus to the operator B, we have

$$\begin{aligned} &r \bigl( \bigl\langle \Phi (A)x,x\bigr\rangle +B-2\bigl\langle \Phi \bigl(A^{\frac{1}{2}}\bigr)x,x \bigr\rangle B^{1/2} \bigr) \\ & \quad\quad {} + r_{0} \bigl( \bigl\langle \Phi \bigl(A^{\frac{1}{2}}\bigr)x,x \bigr\rangle \bigr)B^{ \frac{1}{2}}+\bigl\langle \Phi (A)x,x\bigr\rangle -2\bigl\langle \Phi \bigl(A^{ \frac{3}{4}}\bigr)x,x\bigr\rangle B^{\frac{1}{4}} ) \\ & \quad \leq (1-t)\bigl\langle \Phi (A)x,x\bigr\rangle +tB-B^{t}\bigl\langle \Phi \bigl(A^{1-t}\bigr)x,x \bigr\rangle \\ &\quad \leq R \bigl( \bigl\langle \Phi (A)x,x\bigr\rangle +B-2\bigl\langle \Phi \bigl(A^{ \frac{1}{2}}\bigr)x,x\bigr\rangle B^{\frac{1}{2}} \bigr) -r_{0} \bigl( \bigl\langle \Phi \bigl(A^{\frac{1}{2}}\bigr)x,x\bigr\rangle B^{\frac{1}{2}}+B-2\bigl\langle \Phi \bigl(A ^{\frac{1}{4}}\bigr)x,x \bigr\rangle B^{\frac{3}{4}} \bigr) . \end{aligned}$$

Taking the positive linear map Ψ and the inner product for $y\in {\mathcal{H}}$ with $\Vert y \Vert =1$, we get

$$\begin{aligned} &r \bigl( \bigl\langle \Phi (A)x,x\bigr\rangle +\bigl\langle \Psi (B)y,y\bigr\rangle -2 \bigl\langle \Phi \bigl(A^{\frac{1}{2}}\bigr)x,x\bigr\rangle \bigl\langle \Psi \bigl(B^{1/2}\bigr)x,x \bigr\rangle \bigr) \\ & \quad\quad {} + r_{0} \bigl( \bigl\langle \Phi \bigl(A^{\frac{1}{2}}\bigr)x,x \bigr\rangle \bigr)\bigl\langle \Psi \bigl(B^{\frac{1}{2}}\bigr)x,x\bigr\rangle + \bigl\langle \Phi (A)x,x\bigr\rangle -2\bigl\langle \Phi \bigl(A^{\frac{3}{4}} \bigr)x,x\bigr\rangle \bigl\langle \Psi \bigl(B^{\frac{1}{4}}\bigr)x,x \bigr\rangle \bigr) \\ & \quad \leq (1-t)\bigl\langle \Phi (A)x,x\bigr\rangle +t\bigl\langle \Psi (B)x,x\bigr\rangle - \bigl\langle \Psi \bigl(B^{t}\bigr)x,x\bigr\rangle \bigl\langle \Phi \bigl(A^{1-t}\bigr)x,x\bigr\rangle \\ & \quad \leq R \bigl( \bigl\langle \Phi (A)x,x\bigr\rangle +\bigl\langle \Psi (B)x,x \bigr\rangle -2\bigl\langle \Phi \bigl(A^{\frac{1}{2}}\bigr)x,x\bigr\rangle \bigl\langle \bigl(B^{\frac{1}{2}}\bigr)x,x \bigr\rangle \bigr) \\ & \quad\quad {} -r_{0} \bigl( \bigl\langle \Phi \bigl(A^{\frac{1}{2}}\bigr)x,x \bigr\rangle \bigl\langle \Psi \bigl(B ^{\frac{1}{2}}\bigr)x,x\bigr\rangle +\bigl\langle \Psi (B)x,x\bigr\rangle -2\bigl\langle \Phi \bigl(A^{\frac{1}{4}} \bigr)x,x\bigr\rangle \bigl\langle \Psi \bigl(B^{\frac{3}{4}}\bigr)x,x \bigr\rangle \bigr) . \end{aligned}$$

Now, if we put $x=y$, then we get the desired result. □

### Theorem 9

*Let* $A, B\in \mathcal{B}(\mathcal{H})$ *be positive invertible*. *If* $t\in [0,1]$ *and* Φ, Ψ *are two unital positive linear maps*, *then for any unit vector* $x\in \mathcal{H}$

(i) *for* $0< t\leq \frac{1}{2}$,
  $$\begin{aligned} &2r \bigl( \bigl\langle \Phi (A)x,x\bigr\rangle \nabla \bigl\langle \Psi (B)x,x \bigr\rangle -\bigl\langle \Phi \bigl(A^{\frac{1}{2}}\bigr)x,x\bigr\rangle \bigl\langle \Psi (B)x,x\bigr\rangle ^{1/2} \bigr) \\ & \quad\quad {} + r_{0} \bigl( \bigl\langle \Phi \bigl(A^{1/2}\bigr)x,x \bigr\rangle \bigl\langle \Psi (B)x,x \bigr\rangle ^{1/2}+\bigl\langle \Phi (A)x,x\bigr\rangle -2\bigl\langle \Phi \bigl(A^{3/4}\bigr)x,x \bigr\rangle \bigl\langle \Psi (B)x,x\bigr\rangle ^{1/4} \bigr) \\ &\quad \leq (1-t)\bigl\langle \Phi (A)x,x\bigr\rangle +t\bigl\langle \Psi (B)x,x\bigr\rangle - \bigl\langle \Psi (B)x,x\bigr\rangle ^{t}\bigl\langle \Phi \bigl(A^{1-t}\bigr)x,x\bigr\rangle \\ & \quad \leq 2R \bigl( \bigl\langle \Phi (A)x,x\bigr\rangle \nabla \bigl\langle \Psi (B)x,x \bigr\rangle -\bigl\langle \Phi \bigl(A^{1/2}\bigr)x,x\bigr\rangle \bigl\langle \Psi (B)x,x\bigr\rangle ^{1/2} \bigr) \\ & \quad\quad {} -r_{0} \bigl( \bigl\langle \Phi \bigl(A^{1/2}\bigr)x,x\bigr\rangle \bigl\langle \Psi (B)x,x \bigr\rangle ^{1/2}+\bigl\langle \Psi (B)x,x\bigr\rangle -2\bigl\langle \Phi \bigl(A^{1/4}\bigr)x,x \bigr\rangle \bigl\langle \Psi (B)x,x\bigr\rangle ^{3/4} \bigr) ; \end{aligned}$$
(ii) *for* $\frac{1}{2}< t<1$,
  $$\begin{aligned} &R \bigl( \bigl\langle \Phi (A)x,x\bigr\rangle +\bigl\langle \Psi (B)x,x\bigr\rangle -2 \bigl\langle \Phi \bigl(A^{1/2}\bigr)x,x\bigr\rangle \bigl\langle \Psi (B)x,x\bigr\rangle ^{1/2} \bigr) \\ &\quad\quad {} + r_{0} \bigl( \bigl\langle \Phi \bigl(A^{1/2}\bigr)x,x \bigr\rangle \bigr)\bigl\langle \Psi (B)x,x \bigr\rangle ^{1/2}+\bigl\langle \Phi (A)x,x\bigr\rangle -2\bigl\langle \Phi \bigl(A^{1/4} \bigr)x,x \bigr\rangle \bigl\langle \Psi (B)x,x\bigr\rangle ^{3/4} \bigr) \\ & \quad \leq (1-t)\bigl\langle \Phi (A)x,x\bigr\rangle +t\bigl\langle \Psi (B)x,x\bigr\rangle - \bigl\langle \Psi (B)x,x\bigr\rangle ^{t}\bigl\langle \Phi \bigl(A^{1-t}\bigr)x,x\bigr\rangle \\ & \quad \leq r \bigl( \bigl\langle \Phi (A)x,x\bigr\rangle +\bigl\langle \Psi (B)x,x \bigr\rangle -2\bigl\langle \Phi \bigl(A^{1/2}\bigr)x,x\bigr\rangle \bigl\langle \Psi (B)x,x\bigr\rangle ^{1/2} \bigr) \\ &\quad\quad {} -r_{0} \bigl( \bigl\langle \Phi \bigl(A^{1/2}\bigr)x,x\bigr\rangle \bigl\langle \Psi (B)x,x \bigr\rangle ^{1/2}+\bigl\langle \Phi (A)x,x\bigr\rangle -2\bigl\langle \Phi \bigl(A^{3/4}\bigr)x,x \bigr\rangle \bigl\langle \Psi (B)x,x\bigr\rangle ^{1/4} \bigr) , \end{aligned}$$

*where* $r=\min \{t,1-t\}$, $R=\max \{t,1-t\}$, $r_{0}=\min \{2r,1-2r \}$.

### Proof

The proof of part (ii) is similar to the proof of part (i). Thus we just prove (i). For any positive real number *k* and any unit vector $x\in \mathcal{H}$, we have

22 $$\begin{aligned} &r \bigl( k+\bigl\langle \Psi (B)x,x\bigr\rangle -2\sqrt{k}\bigl\langle \Psi (B)x,x \bigr\rangle \bigr) +r_{0} \bigl( k^{1/2} \bigl\langle \Psi (B)x,x\bigr\rangle ^{1/2} +k-2k ^{3/4}\bigl\langle \Psi (B)x,x\bigr\rangle ^{1/4} \bigr) \\ &\quad \leq (1-t)k+t\bigl\langle \Psi (B)x,x\bigr\rangle -k^{1-t}\bigl\langle \Psi (B)x,x \bigr\rangle ^{t} \\ & \quad \leq R \bigl( k+\bigl\langle \Psi (B)x,x\bigr\rangle -2\sqrt{k}\bigl\langle \Psi (B)x,x \bigr\rangle ^{1/2} \bigr) \\ & \quad\quad {} -r_{0} \bigl( k^{1/2}\bigl\langle \Psi (B)x,x\bigr\rangle ^{1/2}+ \bigl\langle \Psi (B)x,x \bigr\rangle -2k^{1/4} \bigl\langle \Psi (B)x,x\bigr\rangle ^{3/4} \bigr) . \end{aligned}$$

Applying inequality (22) and the functional calculus for the operator *A*, we have

23 $$\begin{aligned} &r \bigl(A+\bigl\langle \Psi (B)x,x\bigr\rangle I_{\mathcal{H}} -2\sqrt{A}\bigl\langle \Psi (B)x,x\bigr\rangle \bigr) \end{aligned}$$

24 $$\begin{aligned} &\quad\quad {} +r_{0} \bigl(A^{1/2}\bigl\langle \Psi (B)x,x\bigr\rangle ^{1/2} +A-2A^{3/4}\bigl\langle \Psi (B)x,x\bigr\rangle ^{1/4} \bigr) \\ &\quad \leq (1-t)A+t\bigl\langle \Psi (B)x,x\bigr\rangle I_{\mathcal{H}}-A^{1-t} \bigl\langle \Psi (B)x,x\bigr\rangle ^{t} \\ & \quad \leq R \bigl(A+\bigl\langle \Psi (B)x,x\bigr\rangle I_{\mathcal{H}}-2\sqrt{A} \bigl\langle \Psi (B)x,x\bigr\rangle ^{1/2} \bigr) \\ &\quad\quad {} -r_{0} \bigl(A^{1/2}\bigl\langle \Psi (B)x,x\bigr\rangle ^{1/2}+ \bigl\langle \Psi (B)x,x \bigr\rangle I_{\mathcal{H}}-2A^{1/4} \bigl\langle \Psi (B)x,x\bigr\rangle ^{3/4} \bigr). \end{aligned}$$

Now, using the unital positive operator Φ and the inner product for $y\in \mathcal{H}$ with $\Vert y \Vert =1$ in inequality (23), we get

$$\begin{aligned} &r \bigl( \bigl\langle \Phi (A)y,y\bigr\rangle +\bigl\langle \Psi (B)x,x\bigr\rangle I_{ \mathcal{H}}-2\bigl\langle \Phi (A)y,y\bigr\rangle ^{1/2}\bigl\langle \Psi (B)x,x \bigr\rangle \bigr) \\ & \quad\quad {} +r_{0} \bigl( \bigl\langle \Phi \bigl(A^{1/2}\bigr)y,y\bigr\rangle \bigl\langle \Psi (B)x,x \bigr\rangle ^{1/2} +\bigl\langle \Phi (A)y,y\bigr\rangle -2\bigl\langle \Phi \bigl(A^{3/4}\bigr)y,y \bigr\rangle \bigl\langle \Psi (B)x,x\bigr\rangle ^{1/4} \bigr) \\ &\quad \leq (1-t)\bigl\langle \Phi (A)y,y\bigr\rangle +t\bigl\langle \Psi (B)x,x\bigr\rangle I _{\mathcal{H}}-\bigl\langle \Phi \bigl(A^{1-t}\bigr)y,y\bigr\rangle \bigl\langle \Psi (B)x,x \bigr\rangle ^{t} \\ & \quad \leq R \bigl( \bigl\langle \Phi (A)y,y\bigr\rangle +\bigl\langle \Psi (B)x,x \bigr\rangle I_{\mathcal{H}}-2\bigl\langle \Phi (A)y,y\bigr\rangle ^{1/2} \bigl\langle \Psi (B)x,x \bigr\rangle ^{1/2} \bigr) \\ & \quad\quad {} -r_{0} \bigl( \bigl\langle \Phi \bigl(A^{1/2}\bigr)y,y\bigr\rangle \bigl\langle \Psi (B)x,x \bigr\rangle ^{1/2}+ \bigl\langle \Psi (B)x,x\bigr\rangle I_{\mathcal{H}}-2\bigl\langle \Phi \bigl(A^{1/4} \bigr)y,y\bigr\rangle \bigl\langle \Psi (B)x,x\bigr\rangle ^{3/4} \bigr) . \end{aligned}$$

Now, putting $y=x$, we get the desired result. □

### Corollary 10

*Let* $A\in \mathcal{B}(\mathcal{H})$ *be positive*, Φ *be a unital positive linear map and* $t\in [0,1]$. *Then for any unit vector* $x\in \mathcal{H}$

(i) *for* $0< t\leq \frac{1}{2}$,
  $$\begin{aligned} &2r\bigl\langle \Phi (A)x,x\bigr\rangle ^{t-\frac{1}{2}} \bigl( \bigl\langle \Phi (A)x,x \bigr\rangle ^{\frac{1}{2}}-\bigl\langle \Phi \bigl(A^{\frac{1}{2}} \bigr)x,x\bigr\rangle \bigr) \\ & \quad\quad {} + r_{0}\bigl\langle \Phi (A)x,x\bigr\rangle ^{t-\frac{1}{2}} \bigl( \bigl\langle \Phi \bigl(A^{1/2}\bigr)x,x\bigr\rangle + \bigl\langle \Phi (A)x,x\bigr\rangle ^{1/2} \\ & \quad\quad {}-2\bigl\langle \Phi \bigl(A^{3/4} \bigr)x,x\bigr\rangle \bigl\langle \Phi (A)x,x\bigr\rangle ^{-1/4} \bigr) \\ & \quad \leq \bigl\langle \Phi (A)x,x\bigr\rangle ^{t}-\bigl\langle \Phi \bigl(A^{t}\bigr)x,x\bigr\rangle \\ & \quad \leq 2R\bigl\langle \Phi (A)x,x\bigr\rangle ^{t-\frac{1}{2}} \bigl( \bigl\langle \Phi (A)x,x\bigr\rangle ^{\frac{1}{2}}-\bigl\langle \Phi \bigl(A^{\frac{1}{2}} \bigr)x,x \bigr\rangle \bigr) \\ & \quad\quad {} - r_{0}\bigl\langle \Phi (A)x,x\bigr\rangle ^{t-\frac{1}{2}} \bigl( \bigl\langle \Phi \bigl(A^{1/2}\bigr)x,x\bigr\rangle + \bigl\langle \Phi (A)x,x\bigr\rangle ^{1/2} \\ & \quad\quad {}-2\bigl\langle \Phi \bigl(A^{1/4} \bigr)x,x\bigr\rangle \bigl\langle \Phi (A)x,x\bigr\rangle ^{1/4} \bigr) ; \end{aligned}$$
(ii) *for* $\frac{1}{2}< t<1$,
  $$\begin{aligned} &2R\bigl\langle \Phi (A)x,x\bigr\rangle ^{t-\frac{1}{2}} \bigl( \bigl\langle \Phi (A)x,x \bigr\rangle ^{\frac{1}{2}}-\bigl\langle \Phi \bigl(A^{\frac{1}{2}} \bigr)x,x\bigr\rangle \bigr) \\ & \quad\quad {} + r_{0}\bigl\langle \Phi (A)x,x\bigr\rangle ^{t-\frac{1}{2}} \bigl( \bigl\langle \Phi \bigl(A^{1/2}\bigr)x,x\bigr\rangle + \bigl\langle \Phi (A)x,x\bigr\rangle ^{1/2} \\ & \quad\quad {}-2\bigl\langle \Phi \bigl(A^{1/4} \bigr)x,x\bigr\rangle \bigl\langle \Phi (A)x,x\bigr\rangle ^{1/4} \bigr) \\ & \quad \leq \bigl\langle \Phi (A)x,x\bigr\rangle ^{t}-\bigl\langle \Phi \bigl(A^{t}\bigr)x,x\bigr\rangle \\ & \quad \leq 2r\bigl\langle \Phi (A)x,x\bigr\rangle ^{t-\frac{1}{2}} \bigl( \bigl\langle \Phi (A)x,x\bigr\rangle ^{\frac{1}{2}}-\bigl\langle \Phi \bigl(A^{\frac{1}{2}} \bigr)x,x \bigr\rangle \bigr) \\ & \quad\quad {} - r_{0}\bigl\langle \Phi (A)x,x\bigr\rangle ^{t-\frac{1}{2}} \bigl( \bigl\langle \Phi \bigl(A^{1/2}\bigr)x,x\bigr\rangle + \bigl\langle \Phi (A)x,x\bigr\rangle ^{1/2}\\ & \quad\quad {}-2\bigl\langle \Phi \bigl(A^{3/4} \bigr)x,x\bigr\rangle \bigl\langle \Phi (A)x,x\bigr\rangle ^{-1/4} \bigr) , \end{aligned}$$

*where* $r=\min \{t,1-t\}$, $R=\max \{t,1-t\}$, $r_{0}=\min \{2r,1-2r \}$.

### Proof

Letting $\Psi =\Phi $ and $B=A$ in Theorem 9, we get the desired inequalities. □

In the next result, we obtain a refinement of inequality (5) for $t\in (0,\frac{1}{2}]$.

### Corollary 11

*Let* $T\in {\mathcal{B}}(\mathcal{H})$ *be positive operator and* $x\in {\mathcal{H}}$ *be a unit vector*. *Then*, *for* $0< t\leq \frac{1}{2}$, *we have*

$$\begin{aligned} &\langle Tx,x \rangle^{t}-\bigl\langle T^{t}x,x\bigr\rangle \\ &\quad \leq 2R\langle Tx,x\rangle^{t-\frac{1}{2}} \bigl( \langle Tx,x \rangle^{\frac{1}{2}}-\bigl\langle T^{\frac{1}{2}}x,x\bigr\rangle \bigr) \\ & \quad\quad {} - r_{0}\langle Tx,x\rangle^{t-\frac{1}{2}} \bigl( \bigl\langle T^{ \frac{1}{2}}x,x\bigr\rangle + \langle Tx,x\rangle^{\frac{1}{2}}-2\bigl\langle T ^{\frac{1}{4}}x,x\bigr\rangle \langle Tx,x\rangle^{\frac{1}{4}} \bigr) \\ & \quad \leq 2R \bigl( \langle Tx,x\rangle^{\frac{1}{2}}-\bigl\langle T^{ \frac{1}{2}}x,x\bigr\rangle \bigr) -r_{0} \bigl( \bigl\langle T^{\frac{1}{2}}x,x \bigr\rangle + \langle Tx,x\rangle^{\frac{1}{2}}-2\bigl\langle T^{\frac{1}{4}}x,x \bigr\rangle \langle Tx,x\rangle^{\frac{1}{4}} \bigr) , \end{aligned}$$

*where* $r=\min \{t,1-t\}$, $R=\max \{t,1-t\}$, $r_{0}=\min \{2r,1-2r \}$.

### Proof

If we replace $\Phi (A)=A$, $A\in \mathcal{B}(\mathcal{H})$ and *t* with $1-t$ in Corollary 10, then we get the desired result. □

## Some new results

In this section, we prove some difference reverse-types of the Hölder and Cauchy–Schwarz inequalities.

### Theorem 12

*Let* $A_{i}, B_{i}\in \mathcal{B}(\mathcal{H})$ ($1\leq i \leq n$) *be positive invertible and* $t\in [0,1]$.

(i) *If* $0< t\leq \frac{1}{2}$, *then*
  $$\begin{aligned} & \Biggl( \sum_{i=1}^{n}A_{i} \Biggr) \sharp_{t} \Biggl( \sum_{i=1}^{n}B_{i} \Biggr) - \Biggl( \sum_{i=1}^{n}A_{i} \sharp_{t}B_{i} \Biggr) \\ &\quad \leq R \Biggl( \sum_{i=1}^{n}A_{i}+ \sum_{i=1}^{n}B_{i}-2\sum _{i=1}^{n}(A _{i}\sharp B_{i}) \Biggr) \\ &\quad\quad {}-r_{0} \Biggl( \sum _{i=1}^{n}(A_{i}\sharp B_{i})+ \sum_{i=1}^{n}B_{i}-2\sum _{i=1}^{n}(A_{i} \sharp_{\frac{3}{4}}B_{i}) \Biggr) \\ &\quad\quad {} -r_{0} \Biggl( \sum_{i=1}^{n}A_{i} \sharp \sum_{i=1}^{n}B_{i}+\sum _{i=1} ^{n}A_{i}-2 \Biggl( \sum _{i=1}^{n}A_{i} \sharp_{\frac{1}{4}}\sum_{i=1} ^{n}B_{i} \Biggr) \Biggr) . \end{aligned}$$
(ii) *If* $\frac{1}{2}< t< 1$, *then*
  $$\begin{aligned} & \Biggl( \sum_{i=1}^{n}A_{i} \Biggr) \sharp_{t} \Biggl( \sum_{i=1}^{n}B_{i} \Biggr) - \Biggl(\sum_{i=1}^{n}A_{i} \sharp_{t}B_{i} \Biggr)\\ &\quad \leq R \Biggl( \sum _{i=1} ^{n}A_{i}+\sum _{i=1}^{n}B_{i}-2 \Biggl( \sum _{i=1}^{n}A_{i}\sharp B_{i} \Biggr) \Biggr) \\ &\quad \quad {} -r_{0} \Biggl( \Biggl(\sum_{i=1}^{n}A_{i} \Biggr)\sharp \Biggl(\sum_{i=1}^{n}B_{i} \Biggr)+\sum_{i=1}^{n}B_{i}-2 \Biggl( \Biggl(\sum_{i=1}^{n}A_{i} \Biggr)\sharp_{\frac{3}{4}}\Biggl( \sum_{i=1}^{n}B_{i} \Biggr) \Biggr) \Biggr) \\ & \quad \quad {} -r_{0} \Biggl( \sum_{i=1}^{n}(A_{i} \sharp B_{i})+\sum_{i=1}^{n}A_{i}-2 \sum_{i=1}^{n}(A_{i} \sharp_{\frac{1}{4}} B_{i}) \Biggr) , \end{aligned}$$
  *where* $r=\min \{t,1-t\}$, $R=\max \{t,1-t\}$ *and* $r_{0}=\min \{2r,1-2r \}$.

### Proof

Taking $A=\operatorname {diag}(A_{1},\ldots,A_{n})$, $B=\operatorname {diag}(B_{1},\ldots,B_{n})$ and $\Phi ( [C_{ij}]_{1\leq i,j\leq n} ) = \sum_{i=1}^{n}C_{ii}$ in equalities (17) and (18), we get the desired inequality. □

Since the function $f(x)=x^{t}$ ($t\in [0,1]$) is an operator concave function, $\sum_{i=1}^{n}w_{i}T_{i}^{t}\leq (\sum_{i=1}^{n}w_{i}T _{i} )^{t}$ for positive operators $T_{i}$ and positive real numbers $w_{i}$ such that $\sum_{i=1}^{n}w_{i}=1$. Now, Theorem 12 yields a reverse of this inequality as follows.

### Example 13

If for positive operators $T_{i}$ ($1\leq i \leq n$), we take $A_{i}=w_{i}I$ and $B_{i}=w_{i}T_{i}$ ($1\leq i \leq n$), in Theorem 12, where $w_{i}$’s are positive real numbers such that $\sum_{i=1}^{n}w_{i}=1$, we obtain the following inequalities:

(i) If $0\leq t\leq \frac{1}{2}$, then
  $$\begin{aligned} \Biggl(\sum_{i=1}^{n}w_{i}T_{i} \Biggr)^{t}-\sum_{i=1}^{n}w_{i}T_{i}^{t} &\leq R \Biggl(I+\sum_{i=1}^{n}w_{i}T_{i}-2 \sum_{i=1}^{n}w_{i}T_{i}^{1/2} \Biggr) \\ & \quad {} -r_{0} \Biggl(\sum_{i=1}^{n}w_{i}T_{i}^{1/2}+ \sum_{i=1}^{n}w_{i}T_{i}-2 \sum_{i=1}^{n}w_{i}T_{i}^{3/4} \Biggr) \\ & \quad {} -r_{0} \Biggl(\Biggl(\sum_{i=1}^{n}w_{i}T_{i} \Biggr)^{1/2}+I-2\Biggl(\sum_{i=1}^{n}w_{i}T _{i}\Biggr)^{1/4} \Biggr). \end{aligned}$$
(ii) If $\frac{1}{2}< t\leq 1$, then
  $$\begin{aligned} \Biggl(\sum_{i=1}^{n}w_{i}T_{i} \Biggr)^{t}-\sum_{i=1}^{n}w_{i}T_{i}^{t} &\leq R \Biggl(I+\sum_{i=1}^{n}w_{i}T_{i}-2 \sum_{i=1}^{n}w_{i}T_{i}^{1/2} \Biggr) \\ &\quad {} -r_{0} \Biggl(\Biggl(\sum_{i=1}^{n}w_{i}T_{i} \Biggr)^{1/2}+ \sum_{i=1}^{n}w_{i}T _{i}-2\Biggl(\sum_{i=1}^{n}w_{i}T_{i} \Biggr)^{3/4} \Biggr) \\ & \quad {} -r_{0} \Biggl(\sum_{i=1}^{n}w_{i}T_{i}^{1/2}+I -2\sum_{i=1}^{n}w_{i}T _{i}^{1/4} \Biggr). \end{aligned}$$

In [18], the Tsallis relative operator entropy $T_{t}(A|B)$ for positive invertible operators *A*, *B* and $0< t\leq 1$ is defined as follows:

$$\begin{aligned} T_{t}(A,B)=\frac{A\sharp_{t}B-A}{t}. \end{aligned}$$

For further information as regards the Tsallis relative operator entropy see [6] and the references therein. In [7, Proposition 2.3], it is shown that for any unital positive linear map Φ the following inequality holds:

25 $$\begin{aligned} \Phi \bigl(T_{t}(A|B)\bigr)\leq T_{t}\bigl( \Phi (A)|\Phi (B)\bigr). \end{aligned}$$

In (25), by similar techniques of Theorem 12, for positive operators $A_{i}$, $B_{i}$ ($i=1,2,\ldots,n$), we have

26 $$\begin{aligned} \sum_{i=1}^{n} \bigl( T_{t}(A_{i}|B_{i}) \bigr) \leq T_{t} \Biggl( \sum_{i=1} ^{n}A_{i}\Big|\sum _{i=1}^{n}B_{i} \Biggr) . \end{aligned}$$

In the next theorem, we show a reverse of inequality (26).

### Theorem 14

*Let* $A_{i}, B_{i}\in \mathcal{B}(\mathcal{H})$ ($1\leq i\leq n$) *be positive invertible and* $t\in (0,1)$.

(i) *If* $0< t\leq \frac{1}{2}$, *then*
  $$\begin{aligned} &T_{t} \Biggl( \sum_{i=1}^{n}A_{i}\Big| \sum_{i=1}^{n}B_{i} \Biggr) -\sum _{i=1} ^{n}\bigl(T_{t}(A_{i}|B_{i}) \bigr) \\ & \quad \leq \frac{1}{t} \Biggl[R \Biggl(\sum_{i=1}^{n}A_{i}+ \sum_{i=1}^{n}B_{i}-2 \sum _{i=1}^{n}(A_{i}\sharp B_{i}) \Biggr) \\ &\quad\quad {}-r_{0} \Biggl(\sum _{i=1}^{n}(A_{i} \sharp B_{i})+ \sum_{i=1}^{n}B_{i}-2\sum _{i=1}^{n}(A_{i} \sharp_{\frac{3}{4}}B_{i}) \Biggr) \\ & \quad\quad {} -r_{0} \Biggl(\sum_{i=1}^{n}A_{i} \sharp \sum_{i=1}^{n}B_{i}+\sum _{i=1} ^{n}A_{i}-2\Biggl(\sum _{i=1}^{n}A_{i} \sharp_{\frac{1}{4}}\sum_{i=1}^{n}B _{i}\Biggr) \Biggr) \Biggr]. \end{aligned}$$
(ii) *If* $\frac{1}{2}< t<1$, *then*
  $$\begin{aligned} &T_{t} \Biggl( \sum_{i=1}^{n}A_{i}\Big| \sum_{i=1}^{n}B_{i} \Biggr) - \sum_{i=1} ^{n}\bigl(T_{t}(A_{i}|B_{i}) \bigr)\\ &\quad \leq \frac{1}{t} \Biggl[R \Biggl(\sum_{i=1}^{n}A _{i}+\sum_{i=1}^{n}B_{i}-2 \Biggl(\sum_{i=1}^{n}A_{i}\sharp B_{i}\Biggr) \Biggr) \\ & \quad \quad {} -r_{0} \Biggl(\Biggl(\sum_{i=1}^{n}A_{i} \Biggr)\sharp \Biggl(\sum_{i=1}^{n}B_{i} \Biggr)+\sum_{i=1}^{n}B_{i}-2 \Biggl(\sum_{i=1}^{n}A_{i} \sharp_{\frac{3}{4}}\sum_{i=1} ^{n}B_{i} \Biggr) \Biggr) \\ &\quad \quad {} -r_{0} \Biggl(\sum_{i=1}^{n}(A_{i} \sharp B_{i})+\sum_{i=1}^{n}A_{i}-2 \sum_{i=1}^{n}(A_{i} \sharp_{\frac{1}{4}} B_{i}) \Biggr) \Biggr]. \end{aligned}$$

### Proof

Applying Theorem 12 for $0< t\leq \frac{1}{2}$, we have

$$\begin{aligned} &T_{t} \Biggl( \sum_{i=1}^{n}A_{i}\Big| \sum_{i=1}^{n}B_{i} \Biggr) -\sum _{i=1} ^{n}\bigl(T_{t}(A_{i}|B_{i}) \bigr) \\ &\quad =\frac{ ( \sum_{i=1}^{n}A_{i} ) \sharp_{t} ( \sum_{i=1} ^{n}B_{i} ) -\sum_{i=1}^{n}A_{i}}{t}-\sum_{i=1}^{n} \frac{A_{i} \sharp_{t}B_{i}-A_{i}}{t} \\ &\quad \leq \frac{1}{t} \Biggl[R \Biggl(\sum_{i=1}^{n}A_{i}+ \sum_{i=1}^{n}B_{i}-2 \sum _{i=1}^{n}(A_{i}\sharp B_{i}) \Biggr)-r_{0} \Biggl(\sum _{i=1}^{n}(A_{i} \sharp B_{i})+ \sum_{i=1}^{n}B_{i}-2\sum _{i=1}^{n}(A_{i} \sharp_{\frac{3}{4}}B_{i}) \Biggr) \\ &\quad\quad {} -r_{0} \Biggl(\sum_{i=1}^{n}A_{i} \sharp \sum_{i=1}^{n}B_{i}+\sum _{i=1} ^{n}A_{i}-2\Biggl(\sum _{i=1}^{n}A_{i} \sharp_{\frac{1}{4}}\sum_{i=1}^{n}B _{i}\Biggr) \Biggr) \Biggr], \end{aligned}$$

whence we get the first inequality. The proof of the second inequality is similar. □

### Remark 15

We can present our results for non-invertible operators; see [6]. It is a direct consequence of the definition of the mean in the sense of Kubo–Ando [11] that $A\sharp_{t} (B+ \varepsilon )$ is a monotone increasing net. Let *B* be a non-invertible operator and $\epsilon >0$. It follows from the set $\{A\sharp_{t}(B+ \epsilon ): \epsilon >0\}$ being bounded above for $0 < \varepsilon <1$ that the limit

27 $$\begin{aligned} A\sharp_{t}B=\lim_{\epsilon \downarrow 0}A \sharp_{t}(B+\epsilon ) \end{aligned}$$

exists in the strong operator topology. So by (27), $A\sharp _{t}B$ exists.
